# Chemogenetic inhibition of dopaminergic neurons reduces stimulus-induced dopamine release, thereby altering the hemodynamic response function in the prefrontal cortex

**DOI:** 10.1162/imag_a_00200

**Published:** 2024-06-21

**Authors:** Cornelia Helbing, Marta Brocka, Alberto Arboit, Michael T. Lippert, Frank Angenstein

**Affiliations:** Functional Neuroimaging Group, Deutsches Zentrum für Neurodegenerative Erkrankungen (DZNE), Magdeburg, Germany; Center for Behavioral Brain Sciences (CBBS), Otto von Guericke University, Magdeburg, Germany; Leibniz Institute for Neurobiology (LIN), Magdeburg, Germany; Medical Faculty, Otto von Guericke University, Magdeburg, Germany

**Keywords:** *in vivo* fast-scan cyclic voltammetry, DREADD, fimbria-fornix, hippocampus, BOLD response

## Abstract

To investigate the effect of endogenously released dopamine on the stimulus-induced blood oxygen level–dependent (BOLD) responses, we used rats expressing inhibitory designer receptors exclusively activated by designer drugs (DREADDs) in neurons of the ventral tegmental area (VTA) and electrically stimulated the fimbria/fornix. This stimulation activates multiple components of the mesolimbic dopamine system, as demonstrated by the BOLD signal changes during functional magnetic resonance imaging (fMRI) and dopamine release in the nucleus accumbens (NAcc) as detected by*in vivo*fast-scan cyclic voltammetry. Activation of inhibitory DREADDs by clozapine*N*-oxide (CNO) significantly reduced stimulus-induced dopamine release and the BOLD response in the NAcc. In contrast, the concurrently induced BOLD response in the medial prefrontal cortex (mPFC) was not significantly reduced after CNO administration, but the hemodynamic response was shifted to the left. Specifically, the Granger causality test showed that the temporal relationship between the BOLD signal changes in the hippocampus and the mPFC, changed. Under control conditions (i.e., in the absence of CNO), the BOLD signal changes in the mPFC and NAcc clearly preceded the BOLD signal changes in the right hippocampus, whereas in the presence of CNO this was only the case for the BOLD signal changes in the NAcc. In the control rats, that is, the rats that received a control virus and thus did not express DREADDs in the VTA, this CNO-mediated effect was not present. Our results indicate that activation of the endogenous dopaminergic system has region-specific effects on the stimulus-induced BOLD responses, so there is no generally applicable fMRI parameter that clearly indicates increased activity of the dopaminergic system.

## 1 Introduction

The mesolimbic dopaminergic system is assumed to play a crucial role for a number of cognitive functions, such as learning and memory formation, reward-related processes, and pain (Robison et al., 2020;Rossato et al., 2009;Serafini et al., 2020;Watanabe & Narita, 2018). Therefore, it would be very useful for cognitive studies to measure noninvasively both the general activity of the mesolimbic system and the activation of its individual components over time. In this way, valuable information could be gathered about when, where, and whether increased dopaminergic transmission is required for the studied cognitive processes. A suitable approach for this endeavor could be functional magnetic resonance imaging (fMRI), as it indirectly maps changes in neuronal activity with high spatial resolution throughout the brain. The initial evidence for an effect of dopaminergic transmission on blood oxygen level–dependent (BOLD) signals came from pharmacological fMRI studies that used either dopamine receptor agonists, nonspecific dopamine-releasing compounds, or dopamine reuptake inhibitors. The findings suggested that dopamine receptor activation modulates BOLD signals in regions with high dopamine receptor levels (Chen et al., 1997;Delfino et al., 2007;Easton et al., 2007,^2009^;Febo et al., 2005;Hewitt et al., 2005;Ireland et al., 2005;Kimura et al., 2023;Taheri et al., 2016). However, these pharmacological fMRI studies cannot properly reflect the temporal aspect of endogenous activation of the dopamine system nor the possible influence of the dopaminergic system on a localized stimulus-induced BOLD response. In one study, the authors tested the effect of dopamine on (visual) stimulus-induced BOLD responses by mimicking increased dopaminergic modulation via systemic application ofl-DOPA (Zaldivar et al., 2014). They reported that the stimulus-induced BOLD responses were reduced by approximately 50%, a decrease they attributed to a dissociation between neuronal and hemodynamic (i.e., blood flow) responses. In another study closely related to the current one, the authors electrically stimulated the perforant pathway during activation (SKF83959) or inhibition (SCH23390) of D1/5-dopaminergic receptors. Under this experimental condition, the average amplitude of the BOLD responses was not significantly altered, but the shape of the hemodynamic response changed (Helbing et al., 2016). Of note, these studies were not able to address the temporal aspect of how a transient activation of the endogenous dopaminergic system would affect the stimulus-induced BOLD responses. Subsequent studies using optogenetic tools to selectively activate in a time-dependent manner dopaminergic neurons in the ventral tegmental area (VTA) have revealed that a temporarily defined neuronal release of dopamine can elicit significant positive BOLD responses (Decot et al., 2016;Ferenczi et al., 2016;Helbing et al., 2016;Lohani et al., 2016). However, these BOLD signal changes were only minor compared with the BOLD responses triggered by, for example, glutamatergic transmission (Brocka et al., 2018). Again, these studies have revealed that endogenous dopamine release can modify BOLD signals but not how and if this would substantially modify the stimulus-induced BOLD responses in regions of the mesolimbic dopamine system.

In the present study, we took advantage of two previous findings: (I) electrical stimulation of the fimbria/fornix activates neurons in the medial prefrontal cortex (mPFC), nucleus accumbens (NAcc), septum, and hippocampus, as well as dopaminergic neurons in the VTA that project to these regions (Helbing & Angenstein, 2020) and (II) a chemogenetic approach (i.e., targeted expression of inhibitory designer receptors exclusively activated by designer drugs [DREADDs] in dopaminergic neurons of the VTA) significantly reduces endogenous dopamine release in the NAcc and mPFC (Halbout et al., 2019). Thus, any changes in the fimbria/fornix stimulation–induced BOLD responses during chemogenetic inhibition of these dopaminergic neurons should indicate the contribution of dopamine to the BOLD response under physiological conditions. That means if a stimulus-related endogenous release of dopamine contributes substantially to a concurrently induced BOLD response in regions of the mesolimbic dopaminergic system, then targeted activation of inhibitory DREADDs should significantly alter these BOLD responses during electrical fimbria/fornix stimulation.

## 2 Materials and Methods

### 2.1 Rats

The rats were cared for and used according to a protocol approved by the Animal Experiment and Ethics Committee and in conformity with European conventions for the protection of vertebrate animals used for experimental purposes as well as institutional guidelines 86/609/CEE (24 November 1986). The experiments were approved by the animal care committee of Saxony-Anhalt (No. 42502-2-1218 DZNE and 42502-2-1705 DZNE) and performed according to the Animal Research: Reporting*In Vivo*Experiments (ARRIVE) guidelines. Male Wistar Han rats were housed individually under a constant temperature (23°C) and maintained on a controlled 12-h day-night cycle with food and tap water available*ad libitum*. A total of 23 rats were used for the*in vivo*experiments: 19 for fMRI (6 of these rats also underwent fast-scan cyclic voltammetry [FSCV] 1 week after fMRI) and 4 were used for*in vivo*electrophysiology.

### 2.2 Virus injection and electrode implantation

#### 2.2.1 Viral vectors and injection surgery

The following viral vectors were used: AAV8-camKIIa-hM4D (Gi)-mcherry, a DREADD virus, and AAV2-camKIIa-mcherry, a control virus. In addition, 3 rats received NaCl solution instead of viral vectors (sham vector). Viral solutions were kindly provided through the Viral Vector Core of the University of North Carolina (https://gtp.med.upenn.edu/vector-core/).

For virus injection, male Wistar Han rats (8–9 weeks old and weighing 280–320 g) were randomly grouped into the DREADD virus, control virus, or saline (NaCl) control groups. Each rat was anesthetized with Nembutal (40 mg/kg, intraperitoneal [i.p.]) and placed in a stereotactic frame. The injection used 10-μL microinjection syringes (Hamilton syringe or Nanofil from World Precision Instruments, Friedberg, Germany) with 33-gauge needles secured to stereotaxic pumps (UMP4 injector; World Precision Instruments).

For all surgeries, 0.65 µL of viral solution (2 × 10E^12^genome copies (gc)/mL) was injected bilaterally in the left and right VTA/substantia nigra (midbrain) (AP −5.6 mm, ML −0.7 mm, and DV 7.2 mm for the first injection and 7.7 mm for the second injection; speed 0.1 µL/min; 5 min rest after each injection). Bilateral injection was used to block/inhibit the neuronal activity of the left and right VTA/substantia nigra during fimbria/fornix stimulation. After injection, bone wax (Butler Schein) was placed over the holes, the wounds were sutured, and the rat was removed from the stereotaxic frame. Each rat also received 3 mL of sterile 0.9% NaCl (subcutaneous) to prevent dehydration. Following surgery, each rat was provided with*ad libitum*food and water and housed individually for a recovery period of 2 weeks.

#### 2.2.2 Implantation of the electrodes

Electrical fimbria/fornix stimulation electrode implantation was performed as described previously (Helbing & Angenstein, 2020). The stimulation electrode was implanted unilaterally in the right hippocampal fimbria by using a rodent stereotaxic frame (Stoelting, Wood Dale, IL, USA). Each rat was anesthetized with Nembutal (40 mg/kg, i.p.) and placed in a stereotactic frame. The bipolar stimulation electrode (114 µm diameter, Teflon-coated tungsten wire, insulated except at the tip; A-M Systems, Science Products GmbH, Hofheim, Germany) was placed unilaterally in the right hippocampal fornix/fimbria fiber tract (AP—1.5 mm, ML 2.6 mm, and DV 2.5–3.3 mm from the dural surface), according to the rat atlas ofPaxinos and Watson (1998). A monopolar recording electrode was lowered into the right nucleus accumbens (r-NAcc) to adjust the correct placement of the stimulation electrode in the right fimbria/fornix (AP 1.7 mm, ML 1.6 mm, and DV 6.5–7.2 mm from the dural surface). In addition, three holes were drilled into the skull and plastic screws were placed to anchor the electrode with dental acrylic (Paladur, Heraeus Kulzer GmbH, Hanau, Germany). The monopolar recording electrode was removed after the stimulation electrode was fixed at the correct position. Following surgery, each rat was provided with*ad libitum*food and water and housed individually for a recovery period of 1 week.

### 2.3 Activation of DREADDs

The expressed DREADD was activated with a low dose of clozapine*N*-oxide (CNO) (0.3 mg/kg, i.p.); the low dose was chosen to minimize the off-target effects of the drug. CNO was dissolved in 0.9% NaCl and injected 30 min before the start of fMRI. Rats injected with the control virus served as a control to test whether CNO alone had an effect on the fMRI or cyclic fast-scan voltammetry results.

### 2.4 Electrical stimulation and fMRI

All fMRI measurements were performed on a 4.7T Bruker Biospec 47/20 animal scanner (free bore of 20 cm) equipped with BGA09 (400 mT/m) gradient system (Bruker BioSpin GmbH, Ettlingen, Germany). A 50-mm Litzcage small animal imaging system (DotyScientific Inc., Columbus, SC, USA) was used to receive the radiofrequency signal.

Each rat was initially anesthetized with isoflurane (1.5%–2.0% in 50:50 N_2_:O_2_, v:v), fixed in the head holder, and connected to the stimulation electrode. Anesthesia was switched to deep sedation by applying medetomidine (Dorbene, Pfizer GmbH, bolus: 50 μg/kg subcutaneous and after 15 min 100 μg/kg per hour subcutaneous (Weber et al., 2006)). Breathing, heart rate, and oxygen saturation were monitored throughout the experiment by using an MRI-compatible pulse oximeter (MouseOX™; Starr Life Sciences Corp., Pittsburgh, PA, USA). Heating was provided from the ventral site.

**Fig. 1.**
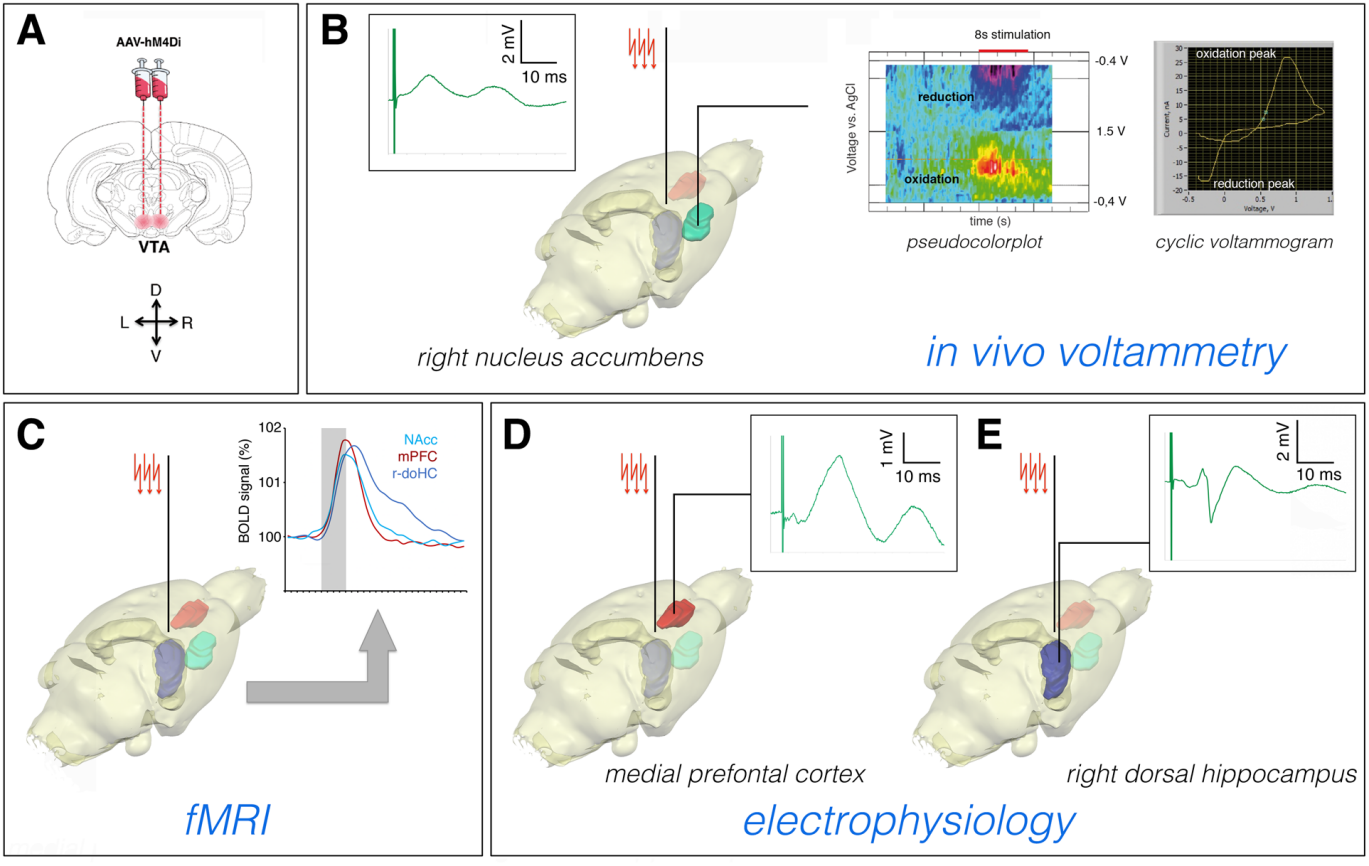
Experimental design. (A) VTA neurons were infected bilaterally with either AAV8-CamKIIa-hM4D (Gi)-mcherry (the DREADD virus) or AAV2-CamKIIa-mcherry (the control virus). (B) Three weeks after virus injection, a stimulation electrode was implanted in the right fimbria/fornix. The correct position of the electrode was adjusted by recording the electrophysiological response in the r-NAcc. The responding electrode was removed after adjusting the stimulation electrode (left box). One week after the second fMRI measurement, a carbon electrode was implanted again in the r-NAcc, and*in vivo*voltammetry was performed to measure stimulus-induced dopamine release in the r-NAcc. (C) The fMRI measurement was performed 1 week after electrode implantation. The same rat was measured two times, after either CNO or NaCl application. Stimulus-induced fMRI responses were simultaneously recorded in all individual VOIs. (D, E) In an additional group of rats,*in vivo*electrophysiology was performed to the measure stimulus-induced neuronal responses in the mPFC (D) and the CA1 region of the dHC (the recordings depict the response to an individual test pulse).

The pulse intensity for the fimbria/fornix stimulation protocol was always set to 250 µA. The pulse width was set at 200 µs, and the mode of stimulation was bipolar in all cases. The stimulation protocol consisted of 10 consecutive stimulation periods given every minute after a 2-min baseline (Fig. 1). Each stimulation period lasted 8 s and contained 8 bursts of high-frequency pulses, that is, 20 pulses with an interval of 10 ms that was given every second. This high-frequency pulse stimulation protocol was used because medetomidine has been shown to inhibit dopamine release only with low-frequency stimulation (Yavich et al., 1997).

Anatomical T_2_*-*weighted spin-echo images were obtained using a rapid acquisition relaxation enhanced (RARE) sequence with the following parameters: repetition time (TR) 4000 ms, time to echo (TE) 15 ms, RARE factor 8, 10 horizontal slices, slice thickness 0.8 mm, field of view (FOV) 37 × 37 mm, matrix 256 × 256, and number of averages 4. The total scanning time was 8 min and 32 s. fMRI was performed using a gradient-echo echo planar imaging (EPI) sequence with the following parameters: TR 2000 ms, TE 24 ms, and matrix 92 × 92. The slice geometry (10 horizontal slices) was identical to the previously obtained anatomical spin-echo-images.

### 2.5 Data processing and analysis

The fMRI data were analyzed with BrainVoyager QX2.6.1 (Brain Innovation, Maastricht, the Netherlands). A standard sequence of preprocessing steps, including slice scan time correction, three-dimensional (3D) motion correction (trilinear interpolation and data reduction using the first volume as a reference), and temporal filtering (full width at half maximum [FWHM] for three data points), was applied to each data set. Images were reconstructed at 128 × 128 voxels per slice and spatially smoothed (Gaussian filter of 1.4 voxels). Functional activation was analyzed by using the correlation of the observed BOLD signal intensity changes in each voxel with a predictor (the hemodynamic response function [HRF]), generated from the given stimulus protocol (see above). To calculate the predictor, the square wave representing the stimulus on and off conditions was convolved with a double gamma HRF (onset 0 s, time to response peak 5 s, and time to undershoot peak 15 s). Based on this, a multi-subject general linear model (GLM) analysis was performed. All significantly activated voxels were converted into volumes of interest (VOIs), from which surface clusters were created and visualized with the BrainVoyager VOI 3D analysis tool. To exclude false positive voxels, only those with a Bonferroni-corrected p < 0.0001 (which corresponds to*t*> 6.11) were considered.

In addition, a VOI analysis was performed, focusing on the following four VOIs: the right dorsal hippocampus (r-dHC), the r-NAcc, the septum, and the dorsal mPFC. All four regions should be directly activated by fimbria/fornix stimulation, and three of them (r-dHC, r-NAcc, and mPFC) are also target regions of dopaminergic projections of the VTA (Morales & Margolis, 2017). The average BOLD time series of all voxels located in one VOI was calculated for each rat by using the VOI analysis tool implemented in the BrainVoyager QX2.6.1 software. Each individual BOLD time series was normalized by using the averaged BOLD signal intensity as 100%. All normalized BOLD time series were averaged and are depicted as the mean ± SD BOLD time series. These mean BOLD time series of individual VOIs were used to calculate the event-related BOLD responses.

The event-related BOLD responses were calculated by measuring the signal intensities starting six frames before stimulus onset (−12 s until 0 s), during stimulus presentation (between 0 and 8 s, which corresponds to 4 frames), and the following 20 frames (10–48 s) after the end of the stimulus. To avoid the confounding effect of putative variations in the baseline BOLD signal intensities on the calculated BOLD response (i.e., BOLD signal_stimulus_/BOLD signal_baseline_× 100%), each BOLD response was related to the BOLD signal intensities of the stimulus over the preceding 12 s.

To gain insight into the HRF, a deconvolution analysis was performed by using a deconvolution design matrix with 10 time points for the last 6 stimulation periods. This deconvolution analysis is part of the BrainVoyager 22.4 analysis software. For the r-dHC and mPFC in each rat, the size of the beta weights was plotted for each time point after stimulation onset and subsequently averaged for each group (DREADD NaCl: n = 5, DREADD CNO: n = 4).

### 2.6 Fscv

A subset of rats that were previously used for the fMRI experiments were also used for FSCV. These rats were anesthetized with urethane (1.6 g/kg i.p.) and placed in the stereotactic frame. A carbon fiber working electrode was lowered into the r-NAcc (AP +1.6 mm and ML +2.2 mm from bregma, DV 7.0–7.5 mm from the dural surface), and recording was started 90 min after implantation of the electrode.

FSCV was performed with polymer-encased carbon fiber electrodes (7 μm diameter, approximately 100–150 μm length; Toray Carbon Fibers America, Inc., Santa Ana, CA, USA) as an acute procedure. The Ag/AgCl reference electrode was prepared from silver wires (0.5 mm diameter, Sigma-Aldrich, St. Louis, MO, USA) that were chloridized in 0.1 M HCl. All cyclic voltammograms were obtained with a triangular waveform (scan rate 10 Hz, resting potential −0.4 V, switching potential 1.5 V, 400 V/s, 1000 samples per scan). Waveform generation and data collection were performed with the Invilog Voltammetric System and Software (Acquisition and Stimulation A&S, Invilog Research Ltd, Kuopio, Finland) and analyzed with the Fast Cyclic Voltammetry Analysis (FSV Analysis, Invilog Research Ltd) tool, which integrates FSCV and displays electrochemical measurements on a base station computer.

*In vivo*changes in the oxidation current recorded with different electrodes (in different rats) cannot be assumed to be equivalent because of the inherent differences in sensitivity between polymer-coated electrodes. Thus, valid comparisons are possible only if the sensitivity of each electrode is calibrated against a standard and the electrochemical data are expressed as standard equivalent values. In the present study, dopamine was used as the standard to calibrate the working electrode sensitivity. Accordingly,*in vivo*changes in the oxidation current are expressed as micromolar dopamine concentrations. Therefore, the peak oxidation currents for dopamine in each voltammogram (at approximately 0.6 V) were converted into concentration from a post-experiment calibration against fresh solutions of 0.1–2 μM dopamine.

Each rat was tested twice, first after NaCl injection and then after CNO injection. NaCl was injected 30 min before the first fimbria/fornix stimulation. One hour after the end of the first stimulation session, CNO was injected i.p., and 30 min later the stimulation was repeated.

To verify expression of DREADDs in the midbrain, eight DREADD virus–injected rats were deeply anesthetized with pentobarbital (50 mg/kg i.p.) and perfused transcardially with 100 mL of 0.1 M phosphate-buffered saline (PBS) followed by 400 mL of 4% paraformaldehyde (PFA) in 0.1 M sodium phosphate (pH 7.4). The brains were removed and post-fixed in 4% PFA for 2 h before being transferred to 30% sucrose in 0.1 M sodium phosphate (pH 7.4) for 48 h at 4°C. The brains were frozen in powdered dry ice and cut on a cryostat to collect coronal sections (40 μm) containing midbrain (approximately −4.8 to −5.6 mm from Bregma).

### 2.7 Statistical analysis

The FSCV datasets were analyzed using SPSS Statistics version 22.0 (IBM Corp., Armonk, NY, USA). A one-way analysis of variance (ANOVA) for unequal variances (Welch’s ANOVA) followed by a Tukey’s post-hoc test was performed to evaluate the dopamine fluctuations during FSCV.

The BOLD time series were analyzed as described previously using paired*t*-tests (Arboit et al., 2022). A two-sample equal variance*t*-test with the Bonferroni correction was used for each time point. For this analysis, p < 0.01 was considered to be significant. A sample size of ≥ 5 rats per group was determined by assuming relevant BOLD signal changes (δ) ≥ 0.5% and a baseline BOLD signal variation (σ) of 0.2% (n = 16/∆^2^, with ∆ = δ/σ) (Schlattmann & Dirnagl, 2010). No anonymizing was done. The temporal relations of the BOLD time series in different VOIs were calculated by using free statistics software for bivariate time series analysis, that is, bivariate Granger causality (Wessa, 2018).

## 3 Results

### 3.1 DREADD expression in the VTA

**Fig. 2.**
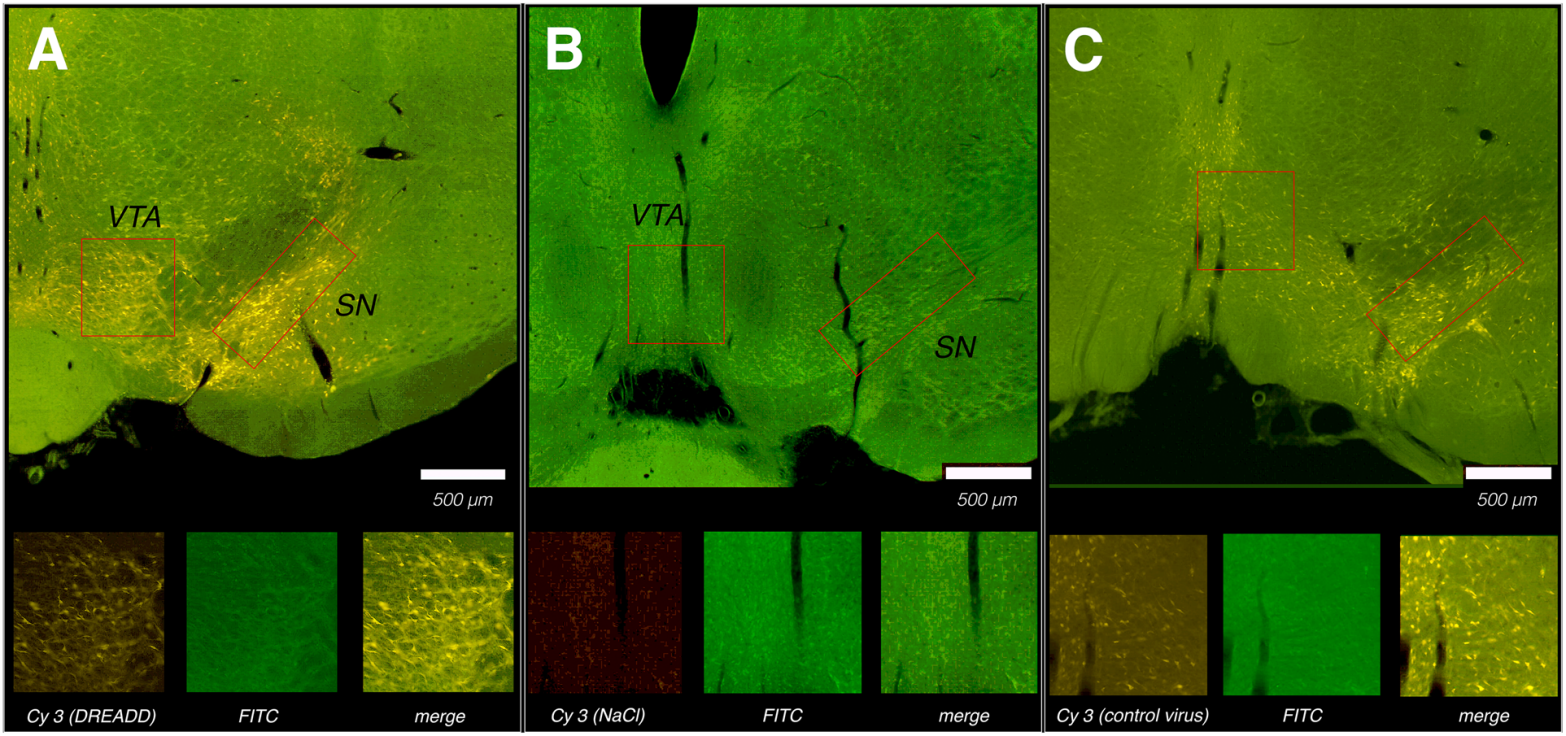
Histological confirmation of DREADD expression in rats that were used for fMRI and*in vivo*voltammetry. (A) Topical injection of AAV8-CamKIIa-hM4D (Gi)-mcherry led to the expression of DREADDs in neurons of the VTA (higher magnification below) and substantia nigra (SN). The presence of DREADDs was confirmed by Cyanine3 (**Cy3**) dye while fluorescein isothiocyanate (**FITC**) autofluorescence served as an anatomical reference. (B) After topical injection of a sham vector solution (NaCl), no Cy3 fluorescence was observed. (C) Topical injection of control AAV2-CamKIIa-mcherry resulted in a similar Cyanine3 (**Cy3**) dye fluorescence.

We confirmed the expression of DREADDs in neurons of the VTA. Fluorescence imaging verified that we had correctly injected the vector and induced effective expression of DREADDs in the VTA (Fig. 2A). There were no nonspecific signals in the rats that received a sham vector injection (i.e., NaCl; control group;Fig. 2B), whereas similar cyanine3 dye (Cy3) fluorescence was detected after injection of the control vector (i.e., AAV2-camKIIa-mcherry;Fig. 2C).

### 3.2 Activation of the mesolimbic system during fimbria/fornix stimulation with a short burst of high-frequency pulses

**Fig. 3.**
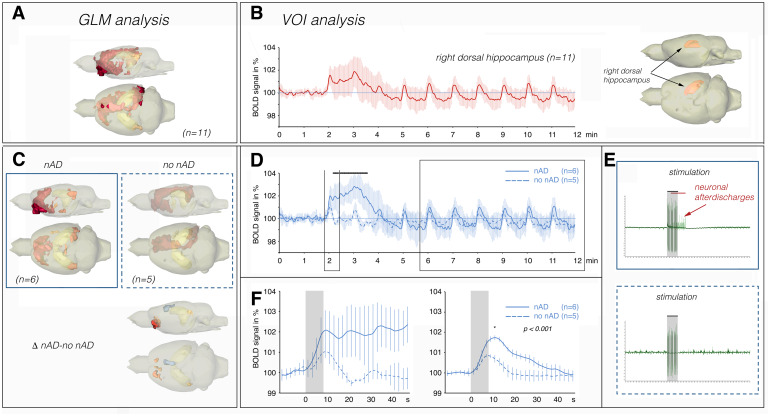
Electrical stimulation of the right fimbria/fornix fibers generates two different BOLD activation patterns. (A) Performing a GLM analysis with the data from all measured rats (n = 11) revealed a widespread BOLD activation pattern (top panel). (B) VOI analysis of all voxels in the r-dHC revealed the presence of strong variability after the first stimulation period when all rats (n = 11) were included in the analysis. (C) Performing the same GLM analysis with two subgroups revealed the presence of two different BOLD activation patterns. The calculation of the contrast between the two groups indicated significantly different clusters of BOLD activation (red clusters: stronger activation for rats with nADs than for rats without nADs; blue clusters: weaker activation for rats with nADs than for rats without nADs; lower panel). (D) The variability after the first stimulation period resulted from the presence of two different BOLD time series: one where the BOLD signals remained elevated after the first stimulation period (solid line), and one where they did not (dashed line). Significant differences between these two BOLD time series are indicated by the black line at the top. (E) Electrophysiological recordings in the r-dHC indicated that the first stimulation period (indicated by the gray box) also caused two neuronal response patterns, one with nADs after cessation of the stimulation (top) and one without nADs (bottom). (F) Comparison of the BOLD responses to the first stimulation period and the averaged responses to the last six stimulation periods. When the first BOLD response did not return to baseline (solid line), the average of the last six BOLD responses was also significantly stronger.

In the first series of experiments, we electrically stimulated the fimbria/fornix of the rats that received the control virus after i.p. injection of 0.9% NaCl (control). Under this condition, consecutive electrical fimbria/fornix stimulation elicited significant BOLD responses in multiple clusters of the rat brain that included parts of the right and left hippocampal formations, the septum, the right-NAcc and the left NAcc, the mPFC, the prelimbic-infralimbic cortex, the right basolateral amygdala, the right piriform cortex, the vertical limb of the diagonal band of Broca, and in the VTA/substantia nigra region (Fig. 3A).

To quantify and compare the BOLD responses in the r-dHC, we performed a VOI analysis and focused on stimulus-related BOLD signal changes in the entire r-dHC (Fig. 3B). Similarly to our previous study (Helbing & Angenstein, 2020), repetitive trains generated two different BOLD responses and time series in the r-dHC (Fig. 3C, D). Specifically, in 6 out of 11 rats, the first stimulation period triggered a significantly prolonged BOLD response in the r-dHC (solid blue line), which was followed by apparently reduced second and third responses before finally returning back to the original value. This phenomenon is in contrast to the other 5 rats, in which all consecutive stimuli triggered uniform and transient responses (dashed blue line inFig. 3D). The existence of two different BOLD time series in the hippocampus has also been observed previously, namely when the perforant pathway was stimulated with the same protocol. Concurrent electrophysiological recordings in the hippocampus revealed that the two different BOLD time series were caused by the presence or absence of neuronal afterdischarges (nADs) after the first stimulation period (Arboit et al., 2021). Therefore, we tested in a separate group of rats whether fimbria/fornix stimulation could also trigger nADs in the hippocampus. Again, the stimulation protocol induced nADs in 2 out of 4 rats (Fig. 3E), suggesting that the two different BOLD time series observed in the r-dHC were also caused by the presence (as reflected by a prolonged first BOLD response) or absence of nADs (as reflected by short first BOLD response) after stimulation.

In addition to the significant difference in the duration of the BOLD response to the first stimulation period, the average size of the BOLD responses was also significantly different. When the first stimulation period caused a prolonged BOLD response, the average BOLD responses were also significantly stronger than in the rats in which the first stimulation period did not cause a prolonged response (Fig. 3F). When performing a GLM analysis for the two groups separately (Fig. 3C), we noted two distinct BOLD activation patterns. Performing a second-level analysis (i.e., calculating the contrast between nADs and no nADs,Fig. 3C) revealed significantly different activation clusters that included the left entorhinal cortex, the left anterior olfactory nucleus and NAcc (nADs > no nADs), and the mPFC (nADs < no nADs).

The reason why the same stimulation triggered nADs (according to the presence of a prolonged first BOLD response) in only a subset of the rats is unclear. It is likely that the stimulation protocol leads to neuronal activation at just the threshold for triggering nADs. The ratio of these two activation patterns was similar in all experimental groups (i.e., control NaCl: 6 rats had nADs and 5 rats had no nADs; control CNO: 7 rats had nADs and 4 rats had no nADs; DREADD NaCl: 5 rats had nADs and 3 rats had no nADs; DREADD CNO: 4 rats had nADs and 4 rats had no nADs) but not identical. To avoid potential problems that could arise from these two different activation patterns, we only used rats in which the first stimulation period elicited nADs in the hippocampus (i.e., a prolonged first BOLD response) for the comparison of the different experimental groups (i.e., NaCl control vs. CNO control and DREADD NaCl vs. DREADD CNO).

**Fig. 4.**
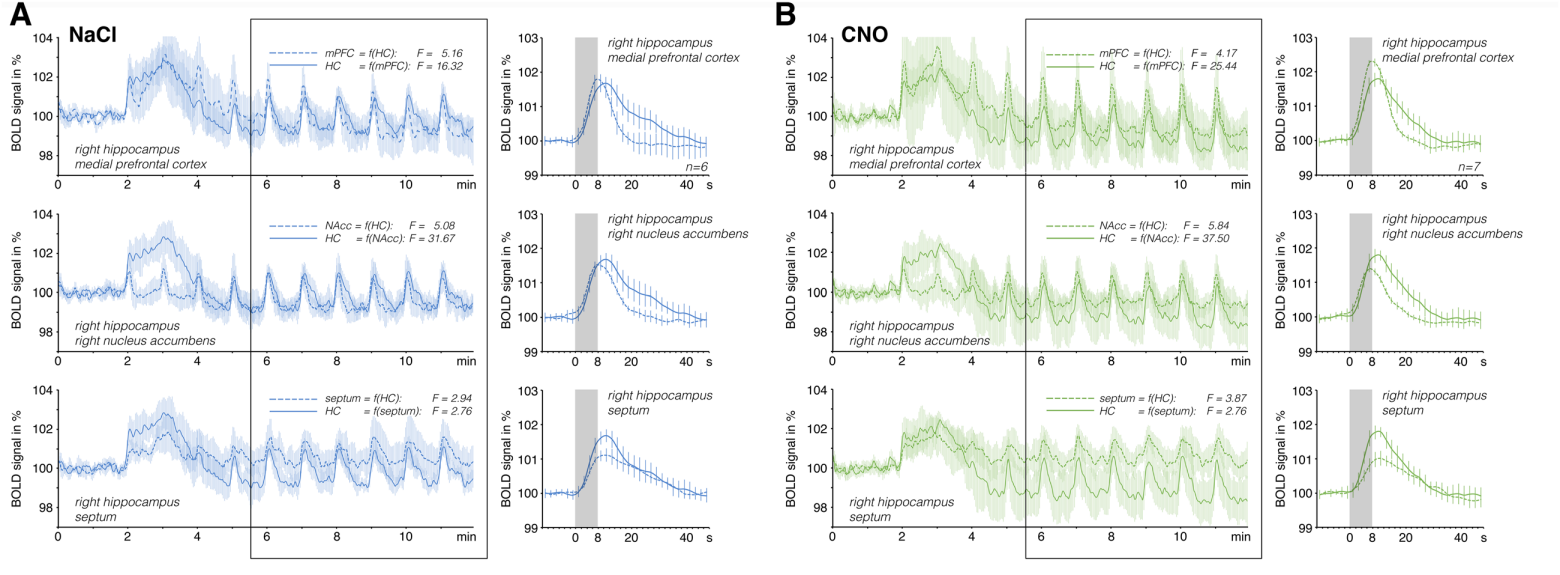
Temporal comparison of the BOLD time series from the different regions in the control rats in the presence of 0.9% NaCl (n = 6; A) and CNO (n = 7; B). According to the Granger causality test, the BOLD time series of the mPFC (top, dashed line) and the r-NAcc (middle, dashed line), but not the septum (bottom, dashed line), preceded the BOLD time series in the r-dHC (solid lines). This is also reflected by comparing the event-related averages of trains 5–10 (right side). Again, the maximum BOLD signal intensity was reached earlier in the mPFC and NAcc than in the r-dHC. The presence of CNO did not affect this temporal relationship.

When the first stimulation period generated a prolonged BOLD response in the r-dHC, there was a prolonged BOLD response in the septum and mPFC, but not in the r-NAcc (Fig. 4,Fig. S1). Separate electrophysiological recordings in the mPFC confirmed that the stimulation could also lead to different neuronal activation patterns there. Again, on the one hand, only immediate pulse-related neuronal reactions were triggered and, on the other hand, there were also longer-lasting increased neuronal activities (Fig. S1). However, it is not clear to what extent the prolonged neuronal activity in the mPFC was locally induced or only propagated from the r-HC.

Next, we compared the time courses of the individual BOLD responses in the four analyzed VOIs. According to the time series, the BOLD signal changes in the mPFC and r-NAcc preceded the BOLD responses in the r-dHC and septum. When we performed a bivariate Granger causality test, both the BOLD time series of the mPFC (F = 16.31, p < 6.6 x 10^-5^) and the r-NAcc (F = 31.67, p < 3.7 x 10^-8^) predicted the BOLD time series of the r-dHC. However, this was not the case for the septum (F = 2.76; p > 0.097). A comparison of the averaged BOLD responses in these four regions shows the different forms of these BOLD responses, which were all triggered simultaneously (Fig. 4A).

**Fig. 5.**
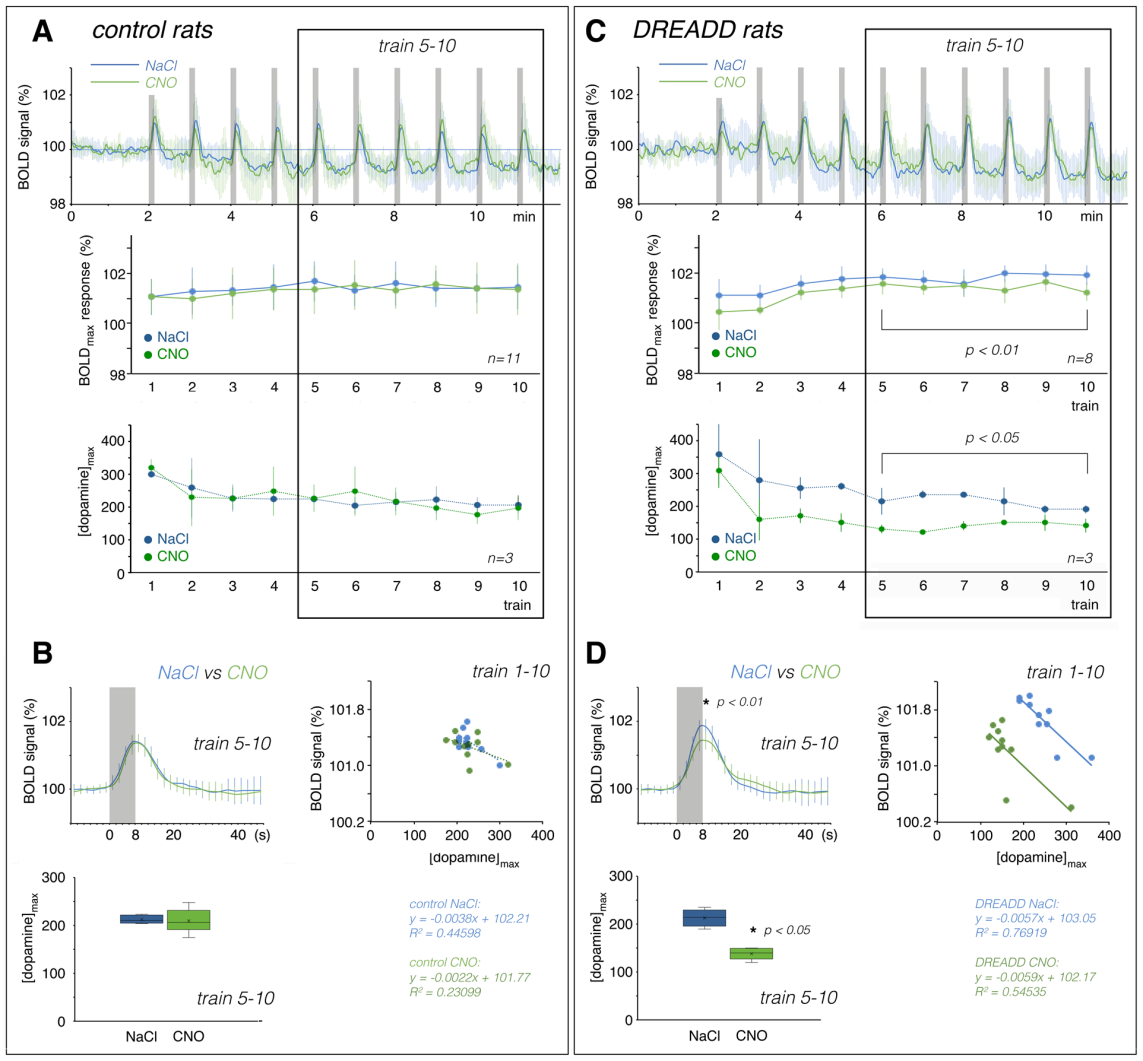
Comparison of the stimulus-related BOLD response and dopamine release in the r-NAcc in the control (A and B) and DREADD-expressing (C and D) rats. (A) In the control rats, the presence of CNO did not affect the magnitude of the BOLD response or dopamine release (NaCl blue line; CNO green line). (B) There was no difference in the average BOLD response (left top) or dopamine release during trains 5–10 (left bottom). Correlating the BOLD response and dopamine release during all 10 consecutive stimulation periods revealed a negative correlation between these two parameters (right side). (C) In the DREADD-expressing rats, the presence of CNO reduced the BOLD responses and dopamine release in the r-NAcc. (D) There was a significant difference in the average BOLD response (left top) and dopamine release during trains 5–10 (left bottom). Correlating the magnitude of BOLD response with the amount of dopamine released during consecutive stimulation (right side) revealed a similar negative correlation between the two parameters in the presence of NaCl and CNO (i.e., the regression line has a similar slope). Thus, the regression line only shifted to the left when CNO activated the inhibitory DREADDs.

In some of these rats, we also measured dopamine release in the r-NAcc during identical fimbria/fornix stimulation. Similarly to the observed BOLD responses in the r-NAcc, we also observed only one pattern of dopamine release with consecutive stimulation. As observed previously (Helbing & Angenstein, 2020), the first stimulation train caused stronger dopamine release than all subsequent stimulation trains, and starting with the fifth stimulation period, stimulus-induced dopamine release stabilized at about 70% of the initial value (Fig. 5A). Overall, when relating the magnitude of the BOLD response with the amount of dopamine released during consecutive stimulation, there was a weak negative correlation (r^2^= 0.443; p = 0.036) between the two parameters (Fig. 5B).

In summary, repeated stimulation of the fimbria/fornix elicited two distinct neuronal response patterns in the r-dHC and mPFC, characterized by the presence or absence of nADs after the first stimulation period. Depending on the presence or absence of nADs, we observed two different BOLD time series in the hippocampus, mPFC, and septum, but not in the r-NAcc, where we observed only one BOLD time series. Likewise, there was only one pattern of dopamine release in the r-NAcc with repeated stimulation, and the amount of dopamine released was rather negatively related to the strength of the BOLD response.

### 3.3 Under the control condition, CNO did not affect the stimulus-induced BOLD responses in all analyzed regions and dopamine release in the r-NAcc

Next, in control rats, we examined whether the presence of 0.3 mg/kg CNO affected stimulus-induced dopamine release and concurrent BOLD response in the r-NAcc. Fimbria/fornix stimulation in the presence of CNO resulted in an almost similar BOLD time series in the r-NAcc (Fig. 5A). Moreover, the presence of CNO did not change stimulus-induced dopamine release in this region (NaCl: 220.2 ± 29.3 nM; CNO: 217.8 ± 47.9 nM; n_rats_= 3; Welch’s ANOVA for unequal variances: F(2, 7.463) = 0.434, p = 0.73;Fig. 5B). The correlation between the amplitude of the BOLD responses and the amount of dopamine released over the course of all successive stimulation periods also showed a weak negative correlation, as previously observed in the presence of NaCl (Fig. 5B). In summary, in control rats, the presence of 0.3 mg/kg CNO had no inhibitory effect on stimulus-induced dopamine release in the r-NAcc and the stimulus-induced BOLD responses in all of the studied brain regions.

### 3.4 Activation of inhibitory DREADDs in neurons of the VTA by CNO reduced stimulation-induced dopamine release and the BOLD response in the r-NAcc

In a second series of experiments, we performed the same stimulation experiments in rats that expressed inhibitory DREADDs in neurons of the VTA. First, we used*in vivo*FSCV to examine whether activation of inhibitory DREADDs in neurons of the VTA by CNO affects stimulus-induced dopamine release in the r-NAcc. Again, in the presence of 0.9% NaCl, the first stimulation train elicited the strongest dopamine release (358.0 ± 53.1 nM); there was less dopamine released during all subsequent fimbria/fornix stimulations after NaCl injection (230.1 ± 8.5 nM;Fig. 5C). In the presence of CNO, the identical fimbria/fornix stimulation protocol led to reduced dopamine release in the r-NAcc compared with the initial dopamine release in the absence of CNO. We observed a small, nonsignificant reduction in dopamine release during the first stimulation period (308.7 ± 30.8 nM), whereas a clear and significant reduction in dopamine release occurred afterward (145.3 ± 4.0 nM; n_rats_= 3; F(2, 17) = 3.956, p < 0.05;Fig. 5C, D).

The presence of CNO also significantly reduced the average amplitude of the BOLD responses during the subsequent stimulation trains (NaCl: 101.85% ± 0.15%; CNO: 101.42% ± 0.13%; p < 0.01;Fig. 5C, D). As a result, the general relationship (i.e., the slope of the calculated regression line) between dopamine release and the magnitude of the BOLD signal did not change, but the position moved to the left (Fig. 5D).

In summary, the presence of 0.3 mg/kg CNO attenuated stimulus-induced dopamine release and the BOLD responses in the r-NAcc in rats that expressed DREADDs in neurons of the VTA, but not in rats that were injected with the control virus.

### 3.5 Activation of inhibitory DREADDs in the VTA modified the stimulus-induced BOLD responses in the mPFC

**Fig. 6.**
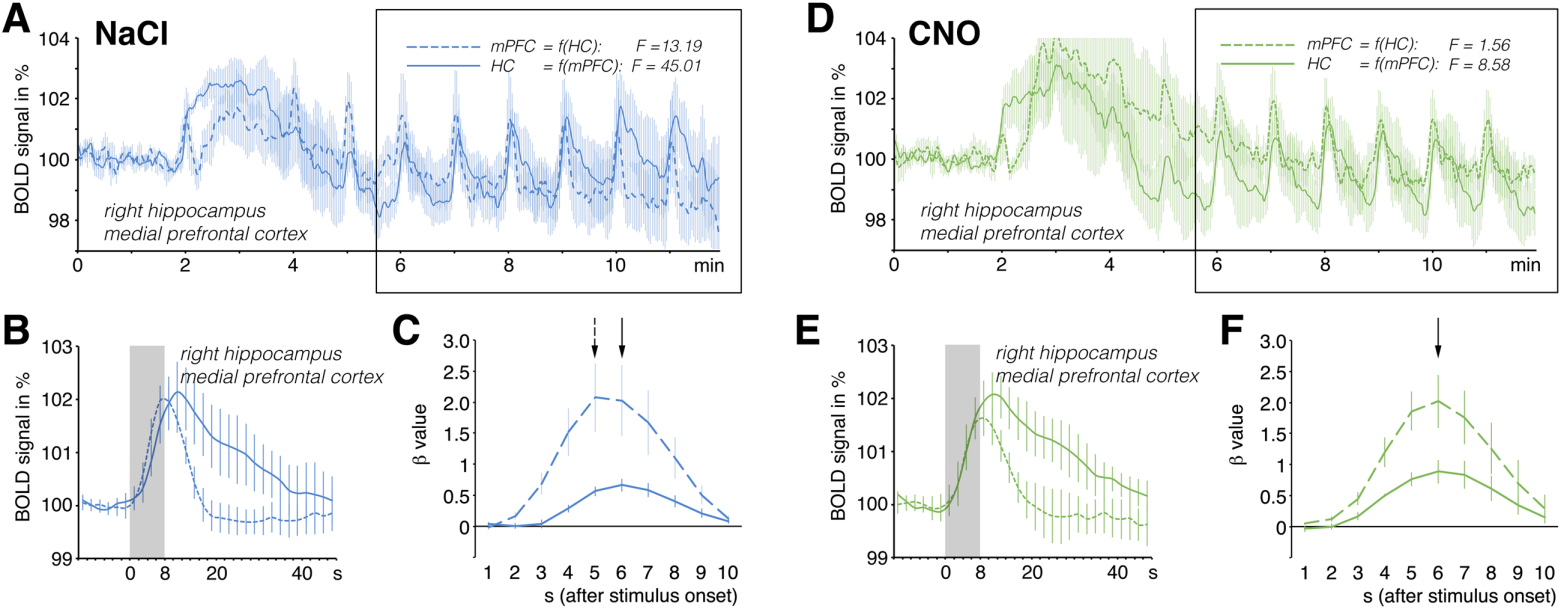
Temporal comparison of the BOLD time series from the r-dHC and the mPFC in the DREADD-expressing rats. (A) In the presence of NaCl (n = 5), the temporal relationship of the BOLD time series was similar to that in the control rats—that is, the BOLD signal changes in the mPFC preceded the BOLD signal changes in the r-dHC. (B) Overlay of the averaged BOLD responses in the r-dHC (solid line) and mPFC (dashed line) revealed an earlier peak of the maximum BOLD response in the mPFC. (C) A deconvolution analysis also revealed the presence of an earlier peak of the β-value in the mPFC (dashed line; the presence of the highest β-value is indicated by the dashed arrow). (D) However, in the presence of CNO (n = 4), the BOLD signals in the mPFC were delayed slightly, so they no longer clearly preceded the BOLD signals in the r-dHC. (E) This is also evident when comparing the event-correlated averages of trains 5–10 (right side) as well as (F) the β-value according to a deconvolution analysis.

Because CNO effectively suppressed the activation of dopaminergic neurons in the VTA of rats that expressed DREADDs in that region, we also measured the stimulus-induced BOLD responses in the other three VOIs in the presence of CNO. Similarly to the control rats, application of CNO had no significant effects on the stimulus-induced BOLD responses in the r-dHC and septum (Fig. 4B, S2). As mentioned previously, in the DREADD-expressing rats, CNO had no effect on the probability of stimulus-induced nADs. Moreover, the presence of CNO did not significantly change the amplitude of the stimulus-induced BOLD responses in the mPFC (Fig. S3); however, it did delay the response. Thus, the BOLD signal changes in the mPFC no longer preceded the BOLD signal changes in the r-dHC (Granger causality test: in the presence of NaCl, F = 45.01, p < 1.7 x 10^-11^; in the presence of CNO, F = 8.58, p < 0.004;Fig. 6A, D). We did not detect a similar shift in the BOLD response in the r-NAcc (Granger causality test: in the presence of NaCl: F = 65.05, p < 1.1 x 10^-14^; in the presence of CNO: F = 77.44, p < 6.1 x 10^-17^) or the septum (Fig. S2). The difference in the shape of the BOLD response (Fig. 6B, E) in the mPFC might result from an altered HRF, as indicated by a deconvolution analysis (Fig. 6C, F).

In summary, in the rats that expressed inhibitory DREADD in neurons of the VTA, the presence of CNO delayed the BOLD response in the mPFC without changing the amplitude. Thus, reduced dopamine release after CNO application had two effects on stimulus-induced BOLD responses: (I) a reduction in the amplitude in the r-NAcc and (II) a change in the shape in the mPFC.

## 4 Discussion

In the current study, we used electrical stimulation of fimbria/fornix fiber to directly activate several target regions of the mesolimbic dopaminergic system (i.e., the hippocampus, mPFC, septum, and NAcc), as well as to activate simultaneously dopaminergic neurons in the VTA that project to these regions. We visualized stimulus-related activation of these target regions by assessing BOLD signal changes in simultaneously performed fMRI. Then, we used a low dose of CNO to activate in one group of rats inhibitory DREADDs that were expressed in neurons of the VTA. As a result, stimulus-induced dopamine release into these structures of the mesolimbic system was significantly reduced (as measured by*in vivo*FSCV in the r-NAcc), and this phenomenon allowed us to determine the effects of stimulus-dependent endogenous dopamine release on the generation of BOLD responses in the target regions mentioned above. Thus, in contrast to our previous work, in which we aimed for a selective time-dependent direct activation of dopaminergic neurons in the VTA by an optogenetic approach (Brocka et al., 2018;Helbing et al., 2016), we reduced the endogenous activation of dopaminergic neurons during stimulation without affecting concurrent transmission of other neurotransmitters (e.g., the glutamatergic and GABAergic systems).

Our main finding is that changes in the endogenous dopamine release affected stimulus-induced BOLD responses in a region- and context-specific manner, that is, we did not observe a generally valid fMRI parameter that clearly correlated with altered dopamine release. The implications of this finding are: (I) There are regional differences, for example, a chemogenetically mediated reduction in stimulus-induced dopamine release resulted in a lower magnitude of BOLD responses in the NAcc (with no detectable difference in the shape of the BOLD response), but simultaneously the shape of the BOLD response in the prefrontal cortex is altered (with no significant change in the amplitude). (II) There are contextual differences in a single region—for example, in the NAcc, repeated stimulation as well as activation of inhibitory DREADDs in dopaminergic neurons attenuate dopamine release. This is associated with either enhanced BOLD responses (during repeated stimulation) or reduced BOLD responses (during activation of inhibitory DREADDs with CNO).

The neurophysiological basis for the region-specific differences remains unknown, but it is consistent with previous results showing that optogenetically induced dopamine release from mesolimbic dopaminergic neurons increases BOLD signals in the striatum (including the NAcc) but not, or to a much lesser extent, in the mPFC (Decot et al., 2016;Lohani et al., 2016). Under this condition, the BOLD signals in the NAcc positively correlate with the amount of dopamine released. As noted previously, activation of D1/5 dopamine receptors by SKF83959 during electrical perforant pathway stimulation changed the shape of the BOLD response most obviously in the mPFC—it accelerated the BOLD response—whereas inhibition of these receptors by SCH23390 delayed the BOLD response (Helbing et al., 2016). This suggests that the effect on the shape of the BOLD response in the mPFC is related to changes in the activity of the dopaminergic system. However, since a number of dopaminergic neurons in the VTA also express the vesicular glutamate transporter 2 (VGLUT2) and thus also release glutamate in their terminals in the nucleus accumbens (Mingote et al., 2019;Stuber et al., 2010;Tecuapetla et al., 2010;Trudeau et al., 2014) and in the prefrontal cortex (Yamaguchi et al., 2011), it is almost impossible to explain the observed changes in BOLD responses with only the effect of a single transmitter system. In particular, because we have also previously observed that inhibition of NMDA receptors by MK801 affects the shape of the BOLD response in the mPFC during stimulation of the perforant pathway (Helbing et al., 2016). Therefore, with our experimental approach, we cannot completely exclude the involvement of glutamatergic neurons for the effect observed here. Interestingly, a subgroup of glutamatergic neurons in the VTA also expresses tyrosine hydroxylase (Th) and thus also releases dopamine in their target regions (Warlow et al., 2024), so that both transmitter systems are again activated together.

Regarding the impact on the magnitude of the BOLD response, it should also be noted that in the r-NAcc, we always observed variable stimulus-induced dopamine release over the course of repeated stimulation under the control conditions (i.e., in the presence of NaCl). There was a strong release during the first stimulation period and a weaker release (approximately 70%) during the later stimulation periods. In contrast, the BOLD responses increased rather than decreased during the repetitive stimulation periods (Fig. 5A). According to this observation, dopamine release would correlate negatively with the stimulus-induced BOLD responses. However, we cannot rule out that repeated stimulation also elicits different neuronal activation patterns. If that were the case, then the measured BOLD responses would reflect these changes rather than the amount of dopamine released. This would again confirm previous observations that the influence of dopamine on the stimulus-induced BOLD response is rather marginal.

In the DREADD-expressing rats, the presence of CNO reduced dopamine release mainly after the initial stimulation period, but there was already an attenuation of the BOLD response during the initial stimulation period (Fig. 5C). Thus, variations in the amount of stimulus-related dopamine release were not unambiguously mirrored by the concurrently induced BOLD responses. Nevertheless, decreased dopamine release after DREADD activation coincided with decreased BOLD responses during all stimulation periods in the r-NAcc, which would be equivalent to a direct relationship between dopamine release and the magnitude of the BOLD response. However, the BOLD response increased during repeated stimulation in both the control and DREADD-expressing rats, and thus the apparent negative correlation between dopamine release and strength of the BOLD response remained. The slope of the correlation line remained the same—it only shifted to the left in the presence of CNO (Fig. 5D). Taken together, these findings indicate that the amount of dopamine released and the associated BOLD responses in the r-NAcc are not really causally related. However, if we assume that the magnitude of the BOLD response mainly reflects the quality/quantity of neuronal activity in this region rather than the amount of dopamine released, this would imply: (I) inhibition of dopaminergic neurons in the VTA of the DREADD-expressing rats by CNO also affects the activity of other neurons (e.g., glutamatergic and GABAergic) projecting to or acting in the r-NAcc or (II) CNO directly inhibits glutamatergic neurons in the VTA and thus reduces the incoming activity in the NAcc. The latter is quite possible given that DREADD expression was under the control of the CaMKIIα promotor, which is not specific for dopaminergic neurons, but rather includes all excitatory neurons (i.e., also glutamatergic). Because glutamatergic neurons of the VTA project directly to the NAcc, but not or to a much lesser extent to the mPFC (Morales & Margolis, 2017;Qi et al., 2016), a CNO-mediated reduction in their activity could primarily affect neuronal activity in the r-NAcc and thus lead to attenuation of the BOLD response in the NAcc but not in the mPFC. Future work with selective expression of DREADDs in dopaminergic neurons (e.g., by using TH-Cre rats) could help to distinguish the role of glutamatergic and dopaminergic transmission from the VTA to the NAcc on control of the BOLD response in the NAcc. However, even with such a more specific approach, the fact described above that a subpopulation of these dopaminergic neurons co-releases glutamate and, conversely, some of the glutamatergic neurons co-release dopamine must be taken into account, which still makes it ambiguous. We found that dopamine release affected the shape of the BOLD response in the mPFC but not in the r-NAcc, although dopamine release was also affected in the latter region. The reason for this effect might be dopamine-dependent control of the HRF in the mPFC. Because we did not simultaneously measure neuronal activity in the mPFC to verify this phenomenon, we used a deconvolution approach to search for an HRF that best matched the observed BOLD response. Although such deconvolution analysis is mainly used to define an HRF in fast event-related fMRI studies, it also points to changes in the HRF in slow event-related fMRI studies. To examine the possible consequences of an altered form of the BOLD response, we conducted a bivariate Granger causality test in two directions. This test does not prove any real causal relationships between the two time courses; it only indicates whether one time course predicts (forecasts) the other, or in our case, whether one time course precedes a second. Granger causality tests have been and are still used in fMRI studies to determine whether neuronal activity in one brain region determines the activity of another region during a particular (e.g., cognitive) task. Based on our observation that the activity of the mesolimbic dopaminergic system significantly modulates the shape of the BOLD response in the mPFC, there may be an apparent appearance or disappearance of dependencies between the mPFC and other regions, according to a Granger causality test, whenever the (cognitive) task affects the activity of the mesolimbic dopaminergic system. Therefore, one should be very cautious in using the Granger causality test in fMRI approaches when there is a possibility that the stimulus may affect the activity of the mesolimbic dopamine system. This view also confirms previous concerns about using the Granger causality test to interpret temporal relationships from a neuroscience perspective (Bielczyk et al., 2019;Stokes & Purdon, 2017).

We should note that we performed the fMRI study in the presence of medetomidine to keep the rats motionless. Stimulus-induced BOLD responses are more pronounced under medetomidine than under isoflurane (Arboit et al., 2023;Krautwald & Angenstein, 2012); however, medetomidine also affects monoaminergic (i.e., noradrenergic and dopaminergic) transmission. Although dopamine release is not impaired by high-frequency pulse stimulation (Yavich et al., 1997), we cannot completely rule out medetomidine-related effects on stimulation-dependent dopamine release. If this were the case, then the effects we described regarding the stimulus-induced BOLD responses should be even stronger in awake rats.

In general, our results suggest that in fMRI studies, increased activity of the mesolimbic dopamine system may have various consequences on the BOLD signals. Specifically, in event-related fMRI, GLM analysis points to slightly stronger BOLD responses, for example, in the NAcc, and a concurrently unchanged maximum BOLD response in the prefrontal cortex would not contradict this phenomenon. On the other hand, dopamine-related changes in the shape of the hemodynamic response may even alter the threshold of significantly activated voxels in the mPFC (when performing a GLM analysis) because the altered BOLD response may deviate from the used predictor (the canonical HRF) to identify these voxels. This may explain previous observations that alterations in the activity of the dopaminergic system coincide with increased BOLD signal variability (Alavash et al., 2018;Garrett et al., 2015). In addition, as mentioned earlier, it may also complicate conclusions about a temporal order in the activity of individual brain regions when a Granger causality test is used to detect them. Thus, researchers should confirm putative activation of the mesolimbic dopamine system by other imaging modalities, such as positron-emission tomography (PET), or use dopamine-sensitive MR contrast agents (Shapiro et al., 2010).

## Supplementary Material

Supplementary Material

## Data Availability

The data sets generated and/or analyzed during the current study are available upon reasonable request from the corresponding author.
