# Supplementary material for: Chemogenetic inhibition of dopaminergic neurons reduces stimulus-induced dopamine release, thereby altering the hemodynamic response function in the prefrontal cortex
Source: Imaging Neurosci (Camb). 2024 Jun 21;2:imag-2-00200. doi: 10.1162/imag_a_00200 (PMC12272248; doi:10.1162/imag_a_00200)
Supplement: Supplementary Material [file imag_a_00200-supp.pdf]

Supplemental figures

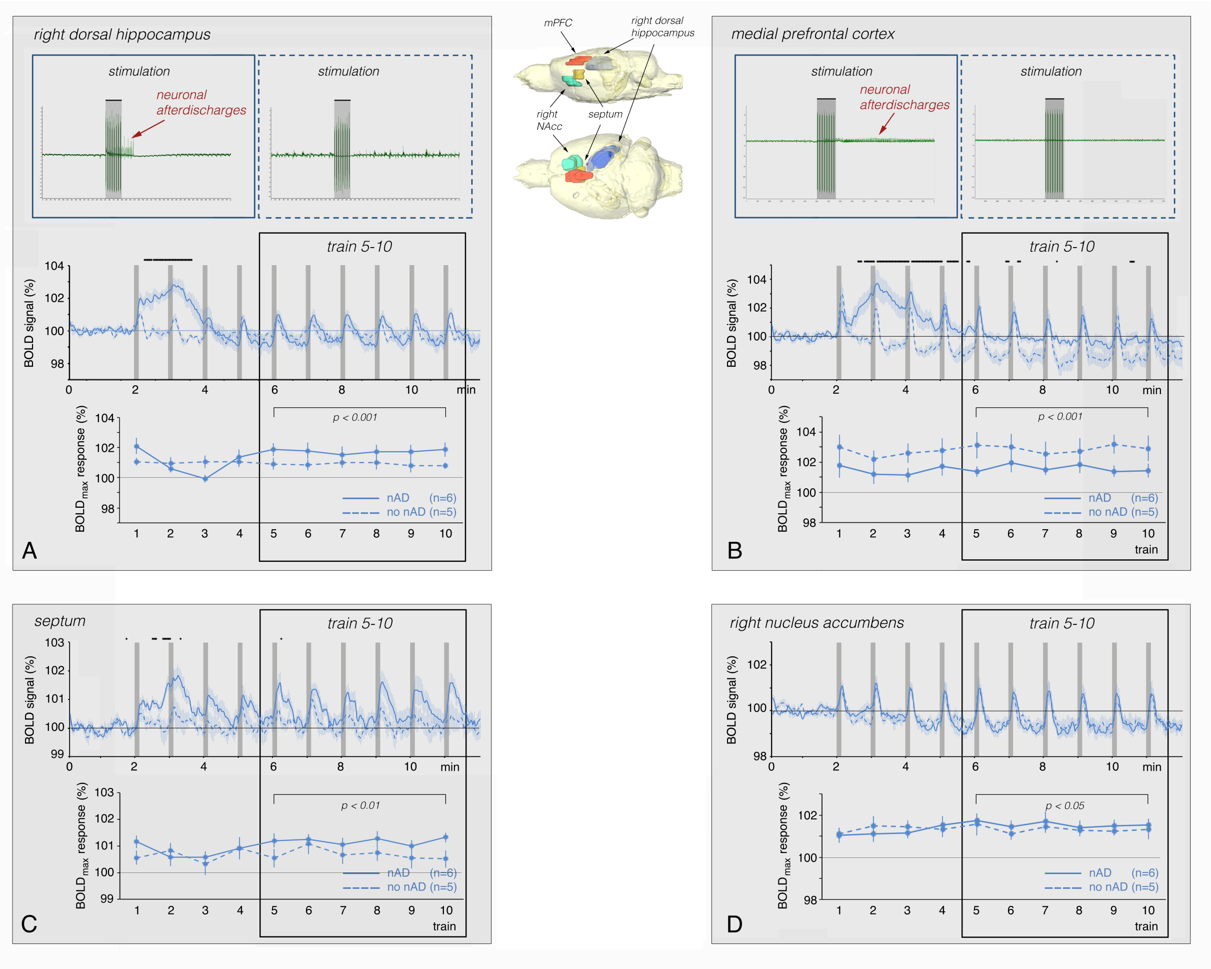

Figure S1

The region-specific BOLD activation pattern during high-frequency pulse stimulation of the fimbria/fornix. To characterize regional specific BOLD responses, the following four VOIs were defined: the mPFC, the r-dHC, the septum, and the r-NAcc. **A** The BOLD time series in the r-dHC. Fimbria/fornix stimulation caused either nADs or no nADs in the hippocampus (top part). Accordingly, two different BOLD time series were observed (nADs, solid line [n = 6], no nADs, dashed line [n = 5], middle part). Periods of significantly different BOLD intensities are indicated by the black line at the top of the graph. The lower graph summarizes the maximum amplitude of the individual BOLD responses during the repetitive stimulation periods. Whereas in the absence of nADs (dashed line) the amplitudes of the BOLD responses remained similar, they varied when the first stimulation period triggered nADs (solid line). Comparison of all amplitudes during late trains (i.e., trains 5–10) revealed a significantly stronger BOLD response when the initial stimulation caused nADs. **B** The BOLD time series in the mPFC. Again, the first stimulation period caused either nADs or no nADs and the two different BOLD time series. In contrast to the r-dHC, the amplitude of the BOLD responses was higher when no nADs where initially elicited. **C** The BOLD time series in the septum. Again, two different BOLD time series were observed, dependent on the presence or absence of nADs in the hippocampus. **D** The BOLD time series in the r-NAcc. In this region, the BOLD time series never showed a prolonged increase after the first stimulation period. Nevertheless, there was a small but significantly stronger BOLD response when nADs were induced in the hippocampus.

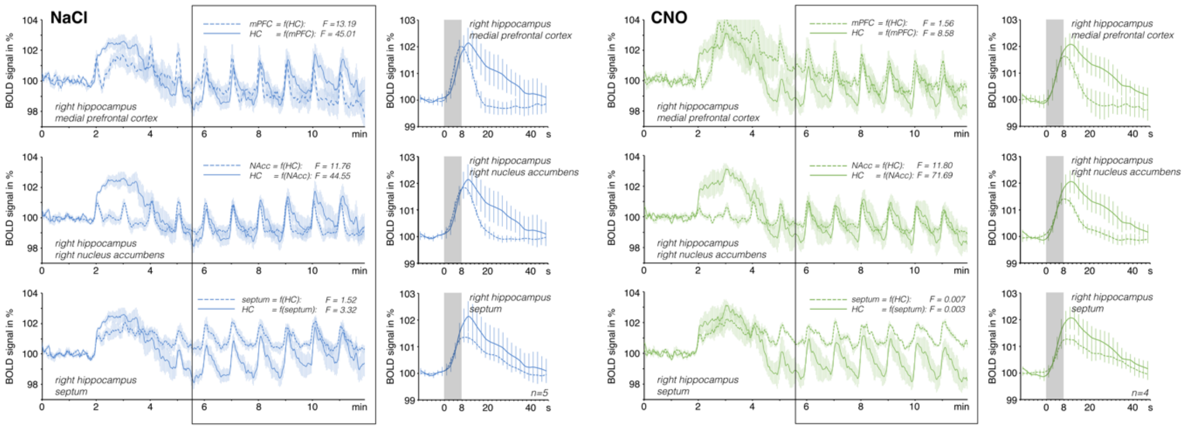

Figure S2

Temporal comparison of the BOLD time series from the four analyzed regions in DREADD-expressing rats. In the presence of NaCl (n = 5), the temporal relationship of the BOLD time series was similar to that in the control rats, that is, the BOLD signal changes in mPFC and r-NAcc preceded BOLD signal changes in r-dHC (see Figure 4). However, in the presence of CNO (n = 4), the BOLD signals in the mPFC were slightly delayed, so they no longer clearly preceded the BOLD signals in the r-dHC. This is also evident when comparing the event-correlated averages of trains 5–10 (right side). A similar shift was not observed in the r-NAcc or septum.

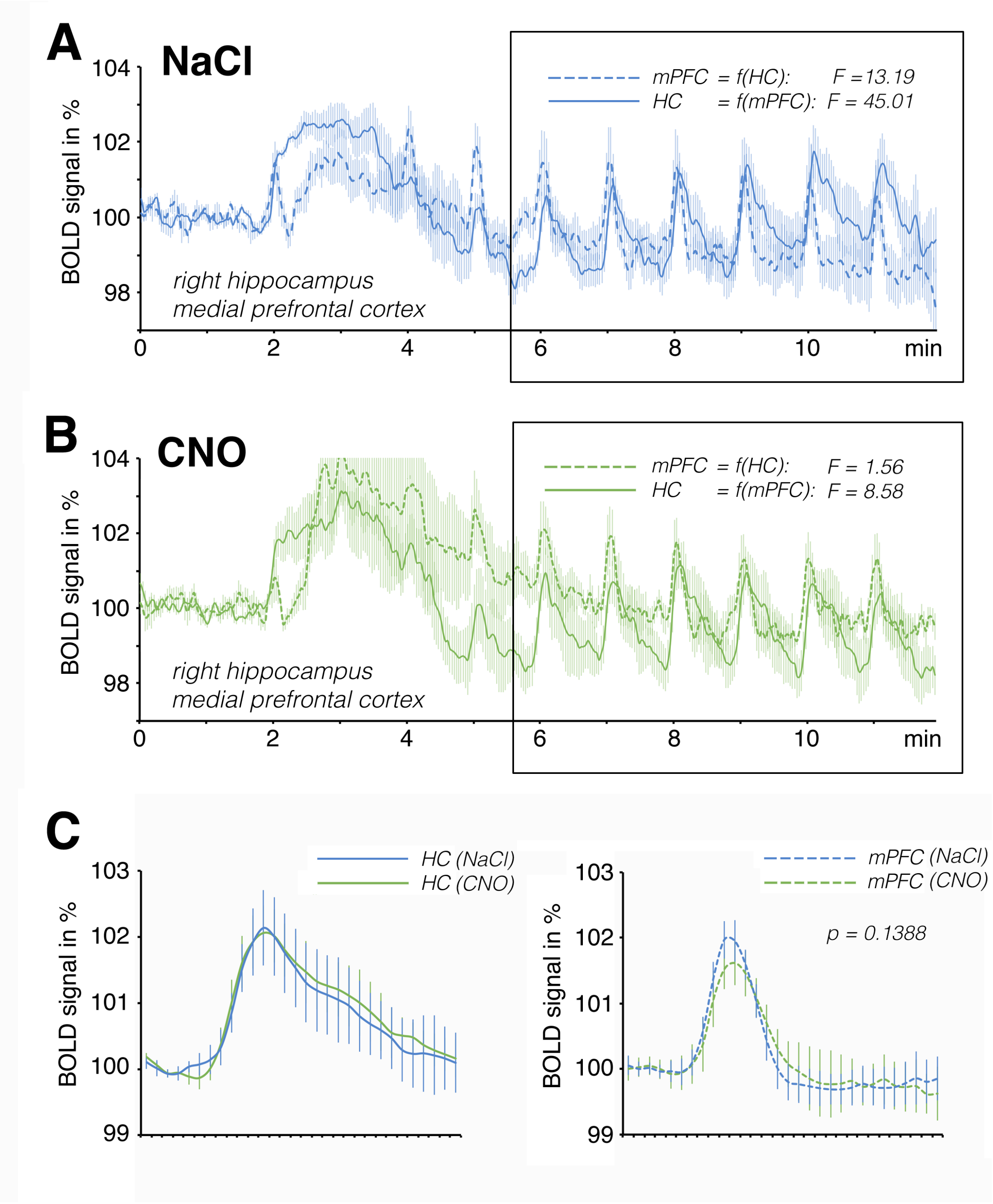

Figure S3

Comparison of the averaged BOLD responses in the r-dHC and mPFC in the rats that expressed DREADDs in the VTA. Overlay of the two BOLD time series after injection of **A** 0.9% NaCl and **B** CNO. **C** Comparison of the averaged event-related BOLD response in the r-dHC when the rats were stimulated in the presence of NaCl (blue line) or CNO (green line). In the hippocampus, the presence of CNO neither affected the amplitude nor the shape of the BOLD response (left side). In contrast, the presence of CNO altered the shape of the BOLD response in the mPFC—that is, the maximum BOLD signal was reached slightly later, although the amplitude did not change significantly (right side).
